# Skin Picking Disorder: A Canadian Retrospective Study of 83 Patients

**DOI:** 10.1177/12034754241303119

**Published:** 2025-01-06

**Authors:** Louis Deschênes, Hélène Veillette

**Affiliations:** 1 Division of Dermatology, Department of Medicine, CHU de Québec-Université Laval, Québec, QC, Canada

**Keywords:** excoriation disorder, psychocutaneous disorder, skin picking disorder, dermatillomania, neurotic excoriations

## Abstract

**Background:**

Skin picking disorder (SPD) is classified as a primary psychodermatologic disorder, in which lesions are self-induced. It is frequently encountered by dermatologists, but the management is still a source of discomfort for the majority.

**Objectives:**

The first objective is to determine the characteristics of the SPD patients in our centre: the demographics, the psychiatric comorbidities, clinical and histopathological characteristics of SPD patients, treatments and follow-up. The second objective is to demonstrate the need for education in the dermatologic community.

**Methods:**

We retrieved and described cases of SPD from January 2011 to December 2022. All inpatients were evaluated at the *Centre Hospitalier Universitaire de Québec – Université Laval.* Qualitative and quantitative data on SPD were collected.

**Results:**

The sample comprised 83 patients, with a mean age of 62 years, and a bimodal distribution. 63.8% were female and lesions were most frequently described as excoriations (27%). Physicians observed picking from multiple body sites, the most common being upper extremities. Only 11% of patients were biopsied. Psychiatric comorbidities were frequent, especially personality traits and disorders (19.2%), and substance-related and addictive disorders (16.8%). Wide variety of treatments were prescribed, including local and supportive care. Only 41% of the sample had medical follow-ups.

**Conclusions:**

This large-scale retrospective assessment of patients diagnosed with SPD broadens the scope of a frequent disorder in dermatology, showing older age patients, unexpected psychiatric comorbidities and inadequate continuity of care. Results highlight the need for a collaborative approach and for frequent reassessments of these patients.

## Introduction

Skin picking disorder (SPD), also known as excoriation disorder or dermatillomania, is classified as a primary psychodermatologic disorder, in which lesions are self-induced and generally without underlying primary skin disease.^1(p128)^ It was first mentioned during the 19th century,^2^ but only officially appeared as a diagnostic in the 5th edition of the American Psychiatric Association’s *Diagnostic and Statistical Manual of Mental Disorders* (DSM-5),^3(p254)^ where SPD was further classified as obsessive-compulsive and related disorders.^4^

According to the DSM-5,^3(p254)^ SPD is a condition in which there is recurrent skin picking resulting in skin lesions, despite repeated attempts to decrease or stop picking. Such behaviour causes significant distress or impairment in important areas of functioning, without having another plausible cause such as a medical condition, physiological effects of a substance, or another mental disorder.^3(p254),5^

SPD has been described as a chronic condition, and varying rates of prevalence in the general population are discussed in the scientific literature, ranging from 1.4% to 5.4%.^6^ This disorder presents a female predominance, with a described age of onset during adolescence.^7^ Even though the comorbidities are essentially psychiatric, this entity is frequently encountered in dermatology given the initial cutaneous presentation.^4^ A better understanding of diagnostic criteria and presentation of SPD is necessary, especially since it has been suggested that dermatologists were sometimes uncomfortable in diagnosing and managing this psychocutaneous disorder.^5,8,9^

The first objective is to determine the characteristics of the SPD patients in our centre: the demographics, the psychiatric comorbidities, clinical and histopathological characteristics of SPD patients, treatments and follow-up. The second objective is to demonstrate the need for education in the dermatologic community, and the importance of multidisciplinarity with mental health services.

## Material and Methods

We obtained Institutional Review Board approval at the *Centre Hospitalier de Québec* for this study, along with the approval of the Director of Professional Services of the Capitale-Nationale and the Ethics Committee of the *Centre Hospitalier de Québec* to access patient files. All procedures contributing to this work comply with the Helsinki Declaration of 1975.

A retrospective chart review was performed for adult patients seen by any provider at *Centre Hospitalier Universitaire de Québec – Université Laval* with the International Classification of Diseases, Clinical Modification (ICD 10-CM) codes L98.1, L20.8, L28.0. These codes were retrieved from hospital discharge summaries from January 1, 2011 to December 31, 2022. With these codes, a total of 315 adult patients were identified. Patients with the following diagnoses were also considered as having SPD for the purpose of this article: “neurotic excoriations,” “excoriation disorder,” “dermatillomania,” “neurodermatitis,”

For these patients, data regarding their demographics, specialists involved, physical exam findings, histopathological findings, psychiatric comorbidities, treatments and follow-up were collected using electronic patient records.

Descriptive characteristics of the sample are presented and were calculated using Microsoft Excel 365.

## Results

### Demographics

A total of 315 patients were identified from hospital discharge summaries as having codes L98.1, L20.8, or L28.0. Of these 315 patients, 83 (n = 83) had a diagnosis of SPD in the retrospective chart review. 53 patients (63.8%) were female, and 36 patients (43.4%) were male. The mean age at diagnosis was 62 years old, with an interval of 24 to 93 years old and a bimodal distribution (54-64, 74-84) (Supplemental Figure 1). Most patients (n = 75) were diagnosed and cared for by dermatologists, and 6 by psychiatrists (Supplemental Figure 2).

### Morphology, Distribution and Histopathological Description

The most common lesion description terms in the patient records were excoriations (n = 49, 27.1%), followed by papules (22.7%), crusts (15.5%), scars (13.3%), plaques (9.9%), erosions or ulcers (6.1%) and nodules (5.5%) (Supplemental Table 1). The most common picking sites were upper limbs (n = 54, 34.2%), followed by trunk (26.6%), lower limbs (25.3%) and head (13.9%) (Supplemental Table 2). Only 9 patients (10.8%) obtained a cutaneous biopsy to further identify the lesions’ cause. Of the 9 biopsy reports, 4 mentioned changes compatible with lichen simplex chronicus. 4 mentioned ulcerations, followed by 2 mentioned inflammation and prurigo nodularis. Erosion, hyperkeratosis and fibrosis were each reported in 1 histopathology report (Supplemental Table 3).

### Psychiatric Comorbidities

The psychiatric comorbidities were collected in the patient files and separated into DSM-5 broader categories^3(p6-7)^ to allow better cohesion in data collection. The majority of SPD patients had at least 1 comorbid psychiatric disorder (n = 51, 61.4%). The most common psychiatric categories were personality traits or disorders (n = 24, 19.2%), substance-related and addictive disorders (16.8%), anxiety disorders (13.6%), neurocognitive disorders (12.8%) and schizophrenia spectrum and other psychotic disorders (11.2%) (Supplemental Table 4). More specifically, all patients with neurocognitive disorders were diagnosed with major neurocognitive disorders in the electronic patient records. Two of these diagnoses were further specified as mixed dementia. Regarding the neurodevelopmental disorders, 2 patients had an intellectual disability, while the others were diagnosed with attention-deficit/hyperactivity disorder.

### Management and Follow-Up

Treatment categories were created to gather all attempted treatments in the 83 SPD patients. Multiple different treatments were tried, the more frequently used being local corticotherapy (n = 40, 24.2%), local antibiotherapy (15.8%), supportive care (13.9%), antihistamines (10.9%), dressings (9.7%) and other local therapies like moisturizing cream, pramoxine and camphor-menthol lotions (9.1%) (Supplemental Table 5). Other attempted treatments are summarized in Supplemental Table 5. Of the 83 SPD patients, the medical follow-up was successfully classified in 81. Most of the patients (n = 48) were dismissed after the diagnosis was made during the first consultation. Thirty-three patients had a follow-up after the diagnosis of SPD.

## Discussion

### Demographics

We conducted a retrospective observational study on a large sample of patients (n = 83) with a diagnosis of SPD. The results showed a mean age at diagnosis of 62 years old (24-93). It is generally accepted that the onset of SPD may occur during adolescence,^1(p134),7,10^ but a trimodal peak of incidence has also been suggested in the literature.^11^ In the latter, the onset would be before 10 years of age, during adolescence and young adulthood and during mid-adulthood (30-45 years old).^12-16^ However, the age at diagnosis tends to be higher than the suspected age of onset.^13^ Mean age at diagnosis varies between as young as 8 years of age to 71 years of age in different recent systematic reviews^17^ and retrospective studies on SPD.^8,18^ Compared to these studies, the mean age and age range of our study are in the upper bound, with a bimodal distribution that is only described in the literature by Ricketts et al,^19^ with a first group in the adolescence period. The incidence of SPD in older adults might be underestimated in scientific literature, partly because this population could be less inclined to answer the electronic-based surveys, which are often the basis of research in SPD.^17^ 63% of the patients in our sample were females, with a ratio woman-to-man of about 3:2. Even though different ratios are suggested in the literature, it generally sits between 3:2 and 3:1 and coincides with the results of this study.^1(p132),7,10,13,18,20^ Most patients were diagnosed by dermatologists (n = 75), in compliance with the notion that SPD is often diagnosed first by dermatologists, considering the frequent initial cutaneous presentation.^21^

### Morphology, Distribution and Histological Description

In prior literature, description terms for the cutaneous lesions of SPD like scars, erosions, lichen simplex chronicus, hypopigmentation, hyperpigmentation, crusts, ulcers, hypertrophic and geometric lesions have been suggested.^1(p134),4,7,12,22^ The population studied had similarly reported lesion morphologies, with terms like excoriations, papules, crusts, scars, plaques, erosions and nodules. In terms of picking sites, the patients with SPD in our study primarily had an involvement of the upper extremities, followed by the trunk, the lower extremities and finally the head. There seems to be a predilection for the accessible sites,^7,23^ particularly the face and the arms in the scientific literature.^4,8,11,12,16,24,25^ Our study goes to show that the location of excoriations can vary greatly in SPD.^1(p134),13,26^ In terms of histopathological studies, the findings in SPD are generally nonspecific, with overlying crusts, erosions and scar tissue.^27^ Hyperkeratosis and hypergranulosis are also used terms in the context of chronic changes like in lichen simplex chronicus and prurigo nodularis.^27^ These findings coincide with the ones of this study, which reported lichen simplex chronicus, inflammation, prurigo nodularis, erosion, hyperkeratosis and fibrosis.

### Psychiatric Comorbidities

In prior literature, SPD has been associated with other psychiatric disorders, notably with major depressive syndrome, obsessive-compulsive disorder and other obsessive-compulsive-related disorders, particularly trichotillomania, and generalized anxiety disorder.^10,18,28-31^ The incidence of these psychiatric comorbidities varies greatly in studies. Some show a higher prevalence of depression,^10,28^ while others have an increased incidence of trichotillomania.^18,30^ Our results differed from these studies. The most common psychiatric categories found in our patients were personality traits or disorders (19.2%), substance-related and addictive disorders (16.8%), anxiety disorders (13.6%) and neurocognitive disorders (12.8%). First, personality traits or disorders are sparsely reported in prior literature, and only a few studies associated personality disorders with SPD.^12,22,31,32^ This notion of comorbidity between SPD and personality disorders or traits could be known and explored, with the goals of raising clinical suspicion and improving the time to diagnosis. Second, the linkage of substance-related and addictive disorders, and SPD is not often reported in the literature. However, some studies associated addictive behaviours with SPD.^31,33^ A clear association with alcohol abuse (39%) was made in a 1999 clinical sample by Wilhelm et al,^31^ before another study in 2014 by Leibovici et al^33^ drew the same conclusion on the subject.^10^ In our group of patients, 16.8% presented a substance-related and addictive disorder. The question raised by this is if these substance-related disorders should be considered comorbidity of SPD or causative, the latter being represented by drugs such as cocaine^34^ and methamphetamines.^35^ A point could be made that by having a substance-related disorder, the patient is possibly not answering the fourth criterion of SPD in the DSM-5.^3(p254)^ Prospective studies are necessary to better explore this interaction. Third, our study showed a high percentage of major neurocognitive disorders in patients at the time of diagnosis. Even though we did not find any studies reporting this association, it is said that neurodegenerative processes could lead up to skin picking behaviours.^17^ In 2016, a case series by Chee et al^36^ identified skin picking behaviours as a prodrome of Parkinson disease, showing the further need to assess this possible association between neurocognitive disorders and SPD and to explore if neurocognitive disorders should be considered part of the SPD’s fourth or fifth criterion in DSM-5.^3(p254)^ Finally, only 3.2% of our patients had a diagnosis of another disorder in the DSM-5 category of obsessive-compulsive and related disorder. Surprisingly, of those 4 patients, none had trichotillomania. Indeed, trichotillomania and other obsessive-compulsive-related disorders are well-known to be associated with SPD as a psychiatric comorbidity.^29,30^ Lifetime prevalence of trichotillomania in SPD patients is said to be 38.8%.^12,37^ We do not have a clear explanation for this impressive difference in statistics. Trichotillomania might have been underdiagnosed at the time of consultation for SPD diagnosis.

### Management and Follow-Up

Non-pharmacological treatments are often viewed as a first-line therapy for SPD.^12^ The cognitive-behavioural therapy (CBT) and more specifically habit-reversal therapy (HRT) are suggested to be the most beneficial psychiatric non-pharmacological treatments.^1(p135),4,5,11,27,38^ These therapies have the potential to improve patients even without pharmacological treatments.^6^ Surprisingly, none of our 83 patients with SPD had mention of CBT or HRT in their electronic records. This major difference with the scientific literature is possibly due to a low accessibility to psychiatric treatments in hospital settings, a low referral rate to psychiatric specialists, and prioritization of psychiatric comorbidities’ treatment during psychiatric consultation. Although few studies mentioned supportive care techniques,^1(p135),17^ 13.9% of our population had been advised to keep nails short, stop manipulation and wear night gloves and long clothing. These techniques are seen as behavioural modifications and can be beneficial for SPD.^17^

Pharmacological treatments should be combined with non-pharmacological therapies as seen above.^4^ No FDA-approved treatments for SPD exist to this date, but many studies on this topic have been conducted in the last 20 years and multiple molecules have been proposed as therapeutic options.^4^ In the present study, multiple topical treatments were prescribed to our population: Topical and intralesional corticotherapy (respectively 24.2% and 1.8%), topical antibiotics (15.8%), dressings, moisturizing cream, pramoxine lotion, camphor lotion and menthol lotion. Some of these treatments were suggested in previous studies,^1(p135)^ but a frequently found concept is that topical therapies do not play a major role in SPD treatment.^8,17,27^ Even though the impact of these topical treatments has not been studied specifically, they seem to be at the basis of the armamentarium for the dermatologists in our study. We think these prescriptions were made mostly to help relieve pruritus and the temptation to manipulate the patients or to treat consequences of SPD, like infections and wounds. However, the mainstay of pharmacological treatments in scientific literature is systemic.^4^ Selective Serotonin Reuptake Inhibitors (SSRIs) are the most studied agents and are often used as first-line therapies.^5,6,38^ Varying results have been found with SSRIs, suggesting that they have better outcomes when concomitant depression, anxiety or obsession-compulsion disorder is present.^4,29^ Fluoxetine, citalopram, escitalopram and fluvoxamine have all been studied with some positive results.^4,38,39^ Some studies suggested that SSRI treatment could be augmented with behavioural therapies and/or antipsychotic drugs,^29^ such as aripiprazole,^27^ and olanzapine.^4^ Still in the antidepressant category, tricyclic antidepressants such as doxepin^1(p135)^ and clomipramine^29^ have been suggested to be beneficial to help with pruritus, sleep disturbances and anxiety.^8^ Since 2016 with Grant et al study,^40^ N-acetylcysteine is thought to reduce skin picking behaviours and is viewed as a good second-line therapy.^12,38,39,41^ Other agents such as topiramate,^12^ lamotrigine,^27^ memantine^17^ and naltrexone^4^ have been studied with inconsistent results. 1st generation antihistamines are generally used to help with pruritus and sleep disturbance.^1(p135),8^ Our results were somewhat different than in prior literature, with a majority of antihistamines and antibiotics used as systemic therapies. The only antidepressant employed was doxepin. Anticonvulsants, such as pregabalin and gabapentin, antibiotherapy, sulfasalazine and systemic corticotherapy were used, without any scientific literature proving their efficacy in SPD. It cannot be forgotten that our population, with a mean age of 62 years old, probably had a lot of medical comorbidities to take into consideration by the clinician in the choice of a pharmacological molecule. It probably changed the approach on certain patients and may have played a role in the low use of systemic agents in our study. Furthermore, major neurocognitive disorder is a comorbidity that seriously impacts the management of SPD. With cognitive decline, patients with dementia experience a lot of challenges associated with the treatment of their other medical conditions.^42^ However, these low percentages might also be attributed to a lack of understanding of SPD and its comorbidities.

The recommended follow-up of patients is not a well-studied aspect of SPD. In our sample, only 41% of patients had at least 1 return appointment. Knowing that SPD is usually chronic,^7^ with exacerbations and remissions over the years,^1(p135)^ a periodic reassessment has been suggested in prior literature.^12^ The absence of follow-up in most of our patients seems to be an inadequate continuity of care, especially knowing that medications and CBT prescribed take months to have a positive impact on the disorder.^11,17^ This lack of reassessment might be due to a misunderstanding of the chronicity and the psychological burden associated with SPD.^4^ However, it remains uncertain if a portion of these patients could have had return appointments outside of hospital settings.

### A Better Understanding

If we circle back to the definition of SPD, it is important to put emphasis on the fourth and fifth criteria, which stipulate that the behaviour is not due to other medical or psychiatric illnesses, or substance use.^3(p254)^ As mentioned above, some of our patients with skin picking behaviours might have been falsely diagnosed as SPD, particularly in cases of substance use and neurocognitive disorders as comorbidities. Along with these uncertain diagnoses, the lack of SSRIs and studied systemic treatments in our study might point towards a misunderstanding of SPD and its needed care. This notion is underscored by a 2010 study by Jafferany et al,^9^ exploring the unfamiliarity in diagnosing and managing psychocutaneous disorders for dermatologists. To help the clinician in his diagnosis, multiple severity assessment scales exist, such as Skin Picking Impact Scale, Skin Picking Impact Survey, Skin Picking Scale-Revised and Milwaukee Inventory for the Dimensions of Adult Skin Picking.^4,27^ Torales et al^12^ also proposed a flow chart for the diagnosis and treatment of excoriation disorder that best summarizes the definition, the psychiatric resources and the necessary follow-ups in patients with SPD. Adequate and up-to-date information on the prescription of psychotropic drugs by dermatologists in SPD can be found in Ha et al 2023 article.^5^

### Limitations

The results of the present study should be considered within the scope of its limitations. These limitations include its retrospective nature and patient selection from tertiary care centre discharge summaries, which limit the generalizability of the findings. The selection of patients only from a tertiary care centre may have overrepresented severe disorders and older patients, more at risk for hospitalization. The retrospective chart review only performed for adult patients might also explain the older mean age of our cohort and the absence of childhood younger peaks of incidence. Finally, provider biases towards patients with existing psychiatric diseases could have led to an overdiagnosis of SPD in this population. Thus, a secondary overestimation of psychiatric comorbidities could have been caused.

## Conclusion

In conclusion, this large-scale retrospective assessment of patients diagnosed with SPD shows that this disorder can present itself in older age patients, unexpected psychiatric comorbidities, large spectrum of treatments and inadequate continuity of care. This study broadens the scope of a frequent disorder in the dermatology’s pool of patients. With the lack of evidence to create management guidelines in SPD,^43^ this retrospective article highlights the need for further studies on pharmacotherapy in different subsets with this psychodermatologic condition. Furthermore, a better understanding of SPD is necessary to ensure these often shamed and embarrassed patients,^44^ affected by this chronic disorder, are treated without counter-transference reactions and with an expression of validation.^16,29^ We believe the necessity for a collaborative approach with a psychiatrist or psychologist must be underscored, along with frequent reassessments of the physical and psychosocial evolution of these patients.^4^

## Supplemental Material

sj-docx-1-cms-10.1177_12034754241303119 – Supplemental material for Skin Picking Disorder: A Canadian Retrospective Study of 83 Patients Supplemental material, sj-docx-1-cms-10.1177_12034754241303119 for Skin Picking Disorder: A Canadian Retrospective Study of 83 Patients by Louis Deschênes and Hélè Veillette in Journal of Cutaneous Medicine and Surgery
