# Supplementary material for: Skin Picking Disorder: A Canadian Retrospective Study of 83 Patients
Source: J Cutan Med Surg. 2025 Jan 6;29(3):268–73. doi: 10.1177/12034754241303119 (PMC12171084; doi:10.1177/12034754241303119)
Supplement: sj-docx-1-cms-10.1177_12034754241303119 – Supplemental material for Skin Picking Disorder: A Canadian Retrospective Study of 83 Patients [file sj-docx-1-cms-10.1177_12034754241303119.docx]

**Supplemental Figures**


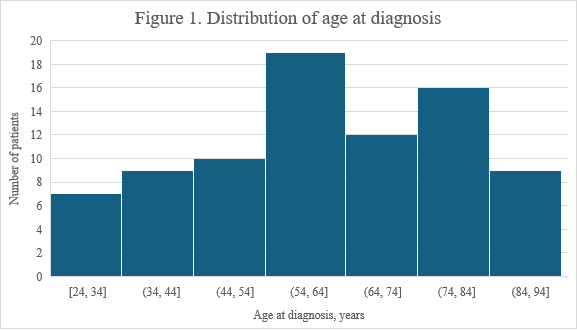


**Supplemental Tables**

| **Morphology** | **n (%)** |
| --- | --- |
| Excoriations | 49 (27.1) |
| Papules | 41 (22.7) |
| Crusts | 28 (15.5) |
| Scars | 24 (13.3) |
| Plaques | 18 (9.9) |
| Erosions/Ulcers | 11 (6.1) |
| Nodules | 10 (5.5) |

Supplemental Table 1. Lesion morphology in the sample group and percentage associated

| **Site of Lesions** | **n (%)** |
| --- | --- |
| Upper Limbs | 54 (34.2) |
| Trunk | 42 (26.6) |
| Lower Limbs | 40 (25.3) |
| Head | 22 (13.9) |

Supplemental Table 2. Lesion distribution in the sample group and percentage associated

Supplemental Table 3. Cutaneous biopsy percentage in the sample group associated with histopathological descriptions and individual percentage

| **Cutaneous Biopsy** | **Histopathological Description** | **n (%)** |
| --- | --- | --- |
| Yes |  | 9 (10.8) |
|  | Lichen Simplex Chronicus | 4 (4.8) |
|  | Ulceration | 4 (4.8) |
|  | Inflammation | 2 (2.4) |
|  | Prurigo Nodularis | 2 (2.4) |
|  | Erosion | 1 (1.2) |
|  | Hyperkeratosis | 1 (1.2) |
|  | Fibrosis | 1 (1.2) |
| No |  | 74 (89.2) |

| **Psychiatric Category Comorbidities** | **n (%)** |
| --- | --- |
| Personality Traits or Disorders | 24 (19.2) |
| Substance-Related and Addictive Disorders | 21 (16.8) |
| Anxiety Disorders | 17 (13.6) |
| Neurocognitive Disorders | 16 (12.8) |
| Schizophrenia Spectrum and Other Psychotic Disorders | 14 (11.2) |
| Depressive Disorders | 9 (7.2) |
| Neurodevelopmental Disorders | 8 (6.4) |
| Obsessive-Compulsive and Related Disorders | 4 (3.2) |
| Somatic Symptom and Related Disorders | 4 (3.2) |
| Trauma- and Stressor-Related Disorders | 4 (3.2) |
| Bipolar and Related Disorders | 1 (0.8) |
| Parasomnias | 1 (0.8) |
| Disruptive, Impulse-Control, and Conduct Disorders | 1 (0.8) |
| Feeding and Eating Disorders | 1 (0.8) |

Supplemental Table 4. Psychiatric category comorbidities, based on the DSM-5, in the sample group and percentage associated

Supplemental Table 5. Attempted management in the sample group and percentage associated

| **Treatments** | **n (%)** |
| --- | --- |
| Local Corticotherapy | 40 (24.2) |
| Local Antibiotherapy | 26 (15.8) |
| Supportive care^1^ | 23 (13.9) |
| Systemic Antihistamines | 18 (10.9) |
| Dressings | 16 (9.7) |
| Other Local Therapies^2^ | 15 (9.1) |
| Systemic Antibiotherapy | 7 (4.2) |
| Phototherapy | 6 (3.6) |
| Antidepressants^3^ | 6 (3.6) |
| Intralesional Corticosteroid Injections | 3 (1.8) |
| Anticonvulsants^4^ | 3 (1.8) |
| Systemic Corticotherapy | 1 (0.6) |
| Systemic Anti-inflammatory^5^ | 1 (0.6) |

Legend:

^1^Supportive care includes keeping nails short, stopping manipulation, long clothing, and night gloves.

^2^Other local therapies include moisturizing cream, pramoxine lotion, camphor and menthol lotion and urea cream.

^3^Antidepressants consist of doxepin.

^4^Anticonvulsants consist of pregabalin and gabapentin.

^5^Systemic anti-inflammatory consists of sulfasalazine.
